# The Nature and Treatment of Diabetes Mellitus

**Published:** 1855-04

**Authors:** F. Headland


*The Nature and Treatment of Diabetes Mellitus.* By Dr. F. Headland.—After a slight general sketch of the symptoms, he proceeded to the inquiry—What is the physiological cause of this abnormal and excessive secretion of glucose, or grape sugar? Three chief theories had been brought forward to account for this:—

1. *Theory of Renal Disorder.*—By Dr. M. Good, and others of his time, it was supposed that the glucose was formed by the kidney in the act of secretion. The author discussed the various alleged morbid conditions of the kidney in diabetes, none of which are known to be constant. The discovery of sugar in the blood and other secretions of diabetes is sufficient to overthrow this theory.

2. *Theory of Saccharine Assimilation*—held by Bouchardat in France, and by the majority of Physicians in England. It supposes that the formation of glucose is due to a deranged digestion or assimilation. Most consider that it is formed in the stomach; others blame the liver. This notion, also, the author disclaimed, arguing that, after a meal on starchy matters, grape sugar may be found in the blood of a healthy man; that it is part of the function of the liver, to form sugar and fat out of albuminous compounds; and that this explanation does not account for the excretion of sugar, for grape sugar given to a healthy man does not pass out in the urine.

3. *Theory of Saccharine Non-Assimilation.*—Supported by Mialhe, Liebig, B. Jones, and others. To this the author gave his own adhesion. It derives confirmation from the experiments of Lehmann, Dumas, and R. D. Thomson. The starch of the food is the chief supporter of the respiratory process. Starch cannot be absorbed without being first changed into dextrine. This is a sort of transition to grape sugar, into which it is all formed in the blood. This grape sugar is not yet in a condition to be oxidized; it is therefore again changed into two atoms of lactic acid, (or some very similar material.) This then combines with oxygen in the blood, supporting the animal heat by its combustion, and forming carbonic acid. These changes require certain agents, probably ferments, to effect them. Supposing they have proceeded as far as glucose, and the agency be wanting which should change this into lactic acid, then the glucose, not being available to the system, is excreted in the urine. The liver attempts to supply the want by forming glucose and fat out of albuminous food. This glucose passes also into the urine. In addition to all this waste, the very tissues are preyed upon to supply fuel for the respiration. The author then discussed at length the subject of treatment, under the following heads:—

A. *Erroneous Plans of Treatment.*

1. Attempts to prevent the formation of glucose.
2. Attempts to hinder the excretive function of the kidney.

The first is a natural process; the second is a healthy provision.

B. *Doubtful Plans of Treatment.*

1. The use of diuretics.
2. Stimulation of the nervous centres, as by strychnia.
3. Treatment directed to the liver.
4. The use of oxidizing agents.

C. *Correct Plans of Treatment.*

*Dietetic Rule.*—To supply, if possible, such articles of food as shall be able, at the same time, to nourish the patient, and to maintain the respiratory combustion without passing through the stage of glucose. (Amongst other things, fat and oils, dry wines, and milk, were recommended.)

*Therapeutic Indications.*

1. To give some remedy that shall seem to be capable of causing the glucose to undergo its normal transformations. (Yeast, rennet, pepsine, &c., were discussed. The author particularly recommended *milk just turned sour*, as containing a decomposing caseine, which transmutes milk sugar into lactic acid. He had advised the use of this remedy in his Essay on the Action of Medicines. It should be used as an article of diet; or it may also be given in enemata, and in warm foot-baths.

2. To replace the urinary secretion by means of diaphoretics and purges. (The author recommended copious sweating, for a physiological reason.)

3. To attend to the general health, and to treat complications.—*London Lancet.*

